# Outcomes of the first global multidisciplinary consensus meeting including persons living with obesity to standardize patient‐reported outcome measurement in obesity treatment research

**DOI:** 10.1111/obr.13452

**Published:** 2022-05-29

**Authors:** Claire E. E. de Vries, Caroline B. Terwee, May Al Nawas, Bart A. van Wagensveld, Ignace M. C. Janssen, Ronald S. L. Liem, Simon W. Nienhuijs, Ricardo V. Cohen, Elisabeth F. C. van Rossum, Wendy A. Brown, Amir A. Ghaferi, Johan Ottosson, Karen D. Coulman, Tarissa B. Z. Petry, Stephanie Sogg, Lisa West‐Smith, Jason C. G. Halford, Ximena Ramos Salas, John B. Dixon, Salman Al‐Sabah, Wei‐Jei Lee, John Roger Andersen, Stuart W. Flint, Maarten M. Hoogbergen, Brooke Backman, Ellen Govers, Nadya Isack, Caroline Clay, Susie Birney, Maureen Gunn, Paul Masterson, Audrey Roberts, Jacky Nesbitt, Riccardo Meloni, Sarah le Brocq, Sandra de Blaeij, Christina Kraaijveld, Floor van der Steen, Bibian Visser, Petra Hamers, Valerie M. Monpellier

**Affiliations:** ^1^ Department of Surgery OLVG Amsterdam The Netherlands; ^2^ Department of Epidemiology and Data Science, Amsterdam UMC Vrije Universiteit Amsterdam, Amsterdam Public Health Research Institute Amsterdam The Netherlands; ^3^ Department of Surgery St. Antonius Hospital Nieuwegein The Netherlands; ^4^ Department of Surgery NMC Royal Hospital Abu Dhabi United Arab Emirates; ^5^ Nederlandse Obesitas Kliniek (Dutch Obesity Clinic) Huis Ter Heide The Netherlands; ^6^ Department of Surgery Groene Hart Hospital Gouda The Netherlands; ^7^ Dutch Obesity Clinic The Hague The Netherlands; ^8^ Department of Surgery Catharina Hospital Eindhoven The Netherlands; ^9^ The Center for Obesity and Diabetes Oswaldo Cruz German Hospital São Paulo Brazil; ^10^ Obesity Centre CGG, Erasmus MC University Medical Center Rotterdam Rotterdam The Netherlands; ^11^ Department of Internal Medicine, Division of Endocrinology, Erasmus MC University Medical Center Rotterdam Rotterdam The Netherlands; ^12^ Department of Surgery, Central Clinical School, Monash University Alfred Hospital Melbourne Victoria Australia; ^13^ Department of Surgery University of Michigan Ann Arbor Michigan USA; ^14^ Department of Surgery, Faculty of Medicine and Health Örebro University Örebro Sweden; ^15^ Bristol Centre for Surgical Research, Population Health Sciences, Bristol Medical School North Bristol NHS Trust Bristol UK; ^16^ Massachusetts General Hospital Weight Center Harvard Medical School Boston Massachusetts USA; ^17^ Department of Surgery, Department of Psychiatry and Behavioral Neuroscience University of Cincinnati College of Medicine Cincinnati Ohio USA; ^18^ School of Psychology University of Leeds Leeds UK; ^19^ Obesity Canada Edmonton Alberta Canada; ^20^ European Association for the Study of Obesity Teddington UK; ^21^ Baker IDI Heart and Diabetes Institute Melbourne Australia; ^22^ Department of Surgery, Jaber Al‐Ahmad Hospital Ministry of Health Kuwait City Kuwait; ^23^ Department of Surgery Min‐Sheng General Hospital Taoyuan Taiwan; ^24^ Faculty of Health and Social Sciences Western Norway University of Applied Sciences Førde Norway; ^25^ Centre of Health Research Førde Hospital Trust Førde Norway; ^26^ Scales Insights, Nexus University of Leeds Leeds UK; ^27^ Department of Plastic Surgery Catharina Hospital Eindhoven The Netherlands; ^28^ Bariatric Surgery Registry Monash University Melbourne Victoria Australia; ^29^ Amstelring and Dutch Knowledge Centre of Dietitians on Obesity (KDOO) Amsterdam The Netherlands; ^30^ Obesity Empowerment Network London UK; ^31^ By‐Band‐Sleeve Study Patient Group London UK; ^32^ European Coalition for People Living with Obesity Dublin Ireland; ^33^ People Living with Obesity Representatives of the S.Q.O.T. Initiative Amsterdam The Netherlands; ^34^ Obesity UK Southport UK; ^35^ KleinePorties Kloetinge The Netherlands

**Keywords:** obesity treatment, patient‐reported outcome measures, patient‐reported outcomes, quality of life

## Abstract

Quality of life is a key outcome that is not rigorously measured in obesity treatment research due to the lack of standardization of patient‐reported outcomes (PROs) and PRO measures (PROMs). The S.Q.O.T. initiative was founded to Standardize Quality of life measurement in Obesity Treatment. A first face‐to‐face, international, multidisciplinary consensus meeting was conducted to identify the key PROs and preferred PROMs for obesity treatment research. It comprised of 35 people living with obesity (PLWO) and healthcare providers (HCPs). Formal presentations, nominal group techniques, and modified Delphi exercises were used to develop consensus‐based recommendations. The following eight PROs were considered important: self‐esteem, physical health/functioning, mental/psychological health, social health, eating, stigma, body image, and excess skin. Self‐esteem was considered the most important PRO, particularly for PLWO, while physical health was perceived to be the most important among HCPs. For each PRO, one or more PROMs were selected, except for stigma. This consensus meeting was a first step toward standardizing PROs *(what* to measure) and PROMs *(how* to measure) in obesity treatment research. It provides an overview of the key PROs and a first selection of the PROMs that can be used to evaluate these PROs.

**Abbreviations**
BARIACT: BARIAtric and metabolic surgery Clinical Trials
BOSS: bariatric and obesity‐specific survey
COMET: Core Outcome Measures in Effectiveness Trials methodology
COS: Core Outcomes Set
COSMIN: COnsensus‐based Standards for the selection of health Measurement INstruments
ICHOM: International Consortium for Health Outcomes Measurement
IWQOL‐Lite: Impact of weight on quality of life‐Lite
OP‐Scale: Obesity‐related Problems Scale
PRO: Patient‐reported outcomes
PROMIS: Patient‐Reported Outcome Measurement Information System
PROMs: patient‐reported outcome measures
QoL: quality of life
QOLOS: quality of life for obesity surgery
S.Q.O.T.: Standardize Quality of life measurement in Obesity Treatment
SF‐36: Short Form‐36
STAR‐LITE: STAndardized Reporting of Lifestyle Weight Management InTerventions to Aid Evaluation

## 1 INTRODUCTION

There is substantial variability in treatment options for obesity, ranging from diet and lifestyle interventions to pharmacological treatment and surgical procedures.^1^, ^2^ With increasing numbers of people undergoing obesity treatment annually, determination of the comparative effectiveness of different treatment options is important.^3^ Although the clinical endpoints of obesity treatments have been well defined and evaluated, the effectiveness of these interventions has not been as adequately assessed from the patient's perspective.^4^, ^5^, ^6^

Patient‐reported outcomes (PROs) directly capture the patient's perspective about the effectiveness of interventions, which are evaluated using PRO measures (PROMs).^7^ Although there are many obesity‐related PROMs, there has been a lack of standardization in the use of these measures.^4^, ^5^, ^6^, ^8^ A systematic review by Coulman et al. demonstrated that in 86 bariatric surgery trials, 1,897 different PROs were measured, with 68 different PROMs.^5^ In weight loss interventions for patients with type 2 diabetes, 20 different PROMs were used in 19 trials.^6^ Both studies were limited in synthesis of PRO data in their meta‐analyses. Moreover, de Vries et al. showed that the measurement properties of many of the PROMs used in bariatric surgery were largely unknown.^8^ Thus, a wide variety of PROMs have been used in obesity treatment research, and many PROMs developed for this population have not been thoroughly validated.

International initiatives, such as the International Consortium for Health Outcomes Measurement (ICHOM) and the Core Outcome Measures in Effectiveness Trials (COMET) initiative, encourage standardization of outcome measurement in clinical practice and clinical trials, respectively.^9^, ^10^ Two studies aimed at standardizing outcome assessment in research of obesity treatment and these studies developed Core Outcomes Sets (COSs) following the COMET methodology.^11^ A COS represents an agreed minimum set of outcomes that should be measured and reported in all clinical trials in a specific area of health.^12^ The BARIAtric and metabolic surgery Clinical Trials (BARIACT) study developed a COS for bariatric and metabolic surgery and overall quality of life (QoL) was one of the nine selected core outcomes.^13^ The STAndardized Reporting of Lifestyle Weight Management InTerventions to Aid Evaluation (STAR‐LITE) study developed a COS for behavioral weight management interventions, and QoL was one of the 24 outcomes prioritized for inclusion in the final COS.^14^ The BARIACT group did not make recommendations for PROMs; in the STAR‐LITE study, they agreed to measure QoL with the EQ‐5D‐5L. However, both initiatives did not obtain consensus on which specific key aspects of QoL should be measured. Because QoL is a broad multidimensional concept that encompasses the emotional, social, and physical well‐being of people's life, it is important to obtain consensus on which of these outcomes matter most to people living with obesity (PLWO) before selecting PROMs.^15^ Additionally, these measures should be selected based on evidence that has been validated with patients undergoing obesity treatment.^11^

Standardization of PRO measurement in obesity treatment research will reduce the heterogeneity of outcomes, enabling the comparison of results across studies and data synthesis. This will improve the quality of evidence used to make well‐informed decisions about obesity treatment. Therefore, building on the two aforementioned consensus efforts, the Standardize Quality of life measurement in Obesity Treatment—S.Q.O.T. initiative—was founded by researchers who focus on the measurement of PROs in obesity treatment. The S.Q.O.T. initiative aims to improve the relevance and consistency of PROs *(what* to measure) and PROMs *(how* to measure) in obesity treatment research. This study reports the results of the first S.Q.O.T. consensus meeting involving PLWO and healthcare providers (HCPs). The objectives of this meeting were twofold: (i) to identify key aspects of QoL (PROs) relevant to be measured in studies on treatment of obesity and (ii) to standardize the future collection of patient‐reported data in such studies by agreeing on preferred PROMs.

## 2 METHODS

This study involved two steps. First, “*what* to measure,” that is, achieving consensus over the relevant PROs in obesity treatment research. Second, “*how* to measure,” that is, achieving consensus on the preferred PROMs to measure the PROs that were considered most relevant. Ethical approval was obtained by the regional institutional review board (Medical research Ethics Committees United, The Netherlands, reference number W21.227).

### 2.1 Systematic review and updated systematic review

The results of a systematic review by de Vries et al. and an update of this review were used as a base for selecting PROs and PROMs.^8^ The first systematic review was performed in 2018 and described the quality of existing PROMs developed and/or validated for QoL measurement in bariatric and body contouring surgery. The update was conducted in 2019 and focused on PLWO undergoing any type of treatment. Only studies with full‐text papers written in English language that aim to describe the development and/or evaluation of measurement properties of PROMs that measure QoL were included. The COnsensus‐based Standards for the selection of health Measurement INstruments (COSMIN) guideline for systematic reviews of measurement instruments was used to evaluate the methodological quality of the included studies and the quality of the PROMs was evaluated by applying quality criteria.^16^ The search for the update was conducted on April 22, 2019. Details of the search are provided in Supporting Information 1. Because the consensus meeting and its prioritization work were conducted in English, only PROMs that were available in the English language and available as full copy were used for the meeting. From the systematic review of de Vries et al., 11 eligible PROMs were identified, and PROs measured with these PROMs were extracted.^8^ The updated search resulted in five additional PROMs, and additional PROs measured with these PROMs were extracted. The results of the updated search are shown in Supporting Information 1. One PROM was brought to our attention by one of the members of the consensus meeting panel.^17^ A total of 25 PROs and 17 PROMs were extracted and discussed in the consensus meeting.

### 2.2 Prioritization surveys

Before the consensus meeting, two prioritization surveys were sent to PLWO and HCPs from North America, South America, Europe, Asia, and Australia: first, to determine which PROs among participants were the most important, and second, to select PROMs that could be eligible for measuring selected PROs. Convenience samples were recruited through national and international healthcare provider federations and patient organizations. The surveys were administered by email with a link to a Web‐based survey (Qualtrics, Provo, UT).^18^ The surveys were sent without an a priori decision about the number of PROs or PROMs that would be included. However, in concordance with previous research, an a priori cut‐off of more than 70% voting “definitely include” or “definitely exclude” was defined to either include or exclude a PRO or PROM for the consensus meeting.^19^, ^20^

The Wilson and Cleary model was used as a conceptual model to provide essential structure to conceptualization of PROs.^21^ This model distinguishes between biological and physiological factors, symptom status, functional status, general health perceptions, and overall quality of life and shows how these outcomes may interrelate. Additionally, the Patient‐Reported Outcome Measurement Information System (PROMIS) conceptual model was used to differentiate between physical, mental, and social aspects of health. PROMIS is a new measurement system for PROs, which is expected to be used more and more worldwide.^22^ PROMIS has developed its own conceptual model of PROs, distinguishing physical, mental, and social aspects of health.^23^

The survey consisted of the 25 PROs extracted from the systematic reviews, and all participants were asked to vote on the PROs (“definitively include,” “possibly include,” or “definitively exclude”) (Supporting information 2). The survey also included a free‐text field to allow the participants to nominate additional PROs not included in the prioritization survey.

The second survey included the PROMs informed by the systematic review described above.^8^ All participants were asked to decide if each PROM was important to be included in the consensus meeting (“definitively include,” “possibly include,” or “definitively exclude”) (Supporting information 2).

### 2.3 Face‐to‐face consensus meeting

A two‐day face‐to‐face consensus meeting was held with PLWO and HCPs. The PLWO were identified through patient organizations or patient representative networks (including participants who participated in the survey), and the HCPs were identified through the professional networks of the organizers. The participants were sent an email invitation describing the objectives of the S.Q.O.T. initiative and meeting. It was ensured that the participants of the consensus meeting were geographically diverse, included a broad range of recognized HCPs and a representative sample of PLWO. The HCPs had expertise in patient‐centered outcomes research, outcome measurement, clinical trials, registries, quality improvement, or healthcare policy. An independent moderator with experience in COS development (CT) led the meeting. The moderator works independently from the S.Q.O.T. initiative and was not involved in the development of any of the PROMs that were included in the meeting. The consensus process was an orientation with formal presentations, a group discussion using nominal group techniques, and Delphi exercises. Nominal group technique and Delphi technique are both established consensus methods that involve a group of stakeholders to generate ideas and establish consensus.^24^, ^25^ Nominal group technique is used to explore ideas in relation to a question to come to an agreement using face‐to‐face discussion and voting, although the Delphi is used to come up with a final decision using anonymous voting and feedback.^26^ The moderator led the group discussion using nominal group techniques. In the Delphi exercise, participants were anonymously asked for their opinion on PROs and PROMs and repolled with controlled and anonymized presentation of results to establish consensus. After each voting round, the combined and stratified analysis (PLWO versus HCPs) of the survey was conducted. The number of rounds in the Delphi exercise was not a pre‐determined, but a dynamic process. All voting was captured electronically and anonymously by using VoxVote.^27^ The organizers (CV, BW, RL, IJ, and VM) and moderator only functioned as facilitators during the consensus meeting and were not permitted to influence the discussions or voting rounds.

#### 2.3.1 Part 1: Orientation

During orientation participants attended a presentation concerning the background and objective of the S.Q.O.T. consensus meeting. Relevant terminology and clarification on the definition of the candidate PROs extracted from the systematic reviews were provided. Furthermore, definitions of measurement properties were explained to enable full participation in the meeting by a methodological expert (CT). In addition, outcomes of the systematic review and the online surveys were presented.

#### 2.3.2 Part 2: What to measure

The results of the prioritization survey on the selection of PROs were first presented and discussed. A group discussion was then held to elicit opinions on the importance of each PRO. The group discussion started with a brainstorm session in which all participants could suggest PROs deemed important. This was to ensure that PROs used in the consensus process were comprehensive from the perspective of different stakeholders (PLWO and HCPs). PROs were generated until saturation was reached. All PROs that were considered important were presented on a list to all participants. The definition of each PRO was also discussed to ensure clarity among all participants and to understand their interpretation. The group discussion informed the list of PROs that were used for the first voting round. After the group discussion, participants were invited to vote on the question “Which three PROs are most relevant to be measured in each research study on obesity treatment?”. The top PROs by average ranking were included in the following voting rounds.

#### 2.3.3 Part 3: How to measure

Subsequently, the PROMs identified in the systematic review and updated version described above that were available for each selected PRO were discussed with the whole group. Participants were provided with a full paper copy of the PROMs for each PRO (labeled according to the outcome of the second prioritization survey), and a paper summary of the measurement properties and feasibility aspects of the PROMs.

Each PROM was discussed separately. First, participants individually assessed the face validity (“the degree as to which the items of an instrument indeed look as though they are an adequate reflection of the construct to be measured”) for each PROM. Second, the group anonymously voted on whether the PROM had sufficient face validity for the specific PRO. Only PROMs that the participants voted as having sufficient face validity were included in the voting round of the respective PRO.

Subsequently, the participants anonymously voted on each of the PROMs separately to the question “Is [PROM name] adequate to measure [PRO] in obesity treatment research?”. On the basis of the COSMIN methodology, professionals were asked whether each PROM (and all of its content) was relevant (“Are the questions relevant to measure [PRO] in persons living with obesity?”) and comprehensive (“With regard to [PRO] in persons living with obesity, are there any key aspects missing?”).^28^ In addition to the relevance and comprehensiveness, PLWO were also asked whether the PROMs were comprehensible (“Are the questions and response options understandable?”).^28^ An a priori cut‐off of more than 70% of the participants or more than 70% of the PLWO was needed to endorse a PROM to be included for that specific PRO. This cut‐off has been considered appropriate in similar consensus studies.^19^, ^20^ Participants involved in the development of one of the eligible PROMs were excluded from the voting round.

## 3 RESULTS

### 3.1 The face‐to‐face consensus meeting

On September 1 and 2 in 2019, 35 participants from North America, South America, Europe, Asia, and Australia participated in the face‐to‐face consensus meeting in Amsterdam, the Netherlands. The participants included 16 PLWO and 19 HCPs (surgeons, endocrinologists, other physicians specialized in obesity treatment, dieticians, physiotherapists, and clinical psychologists). Three participants canceled just before the meeting due to personal reasons (*n* = 1) or flight cancelation (*n* = 2).

### 3.2 *What* to measure

The prioritization survey to rank the PROs was completed by 111 PLWO and HCPs. The following six PROs were selected for discussion in the consensus meeting (>70% “definitely include”): physical health, psychological health, physical symptoms, mental health, self‐esteem, and pain. The results of this survey are shown in Table 1 and Supporting Information 3.

**TABLE 1:** Patient‐reported outcomes (PROs) that were endorsed (>70% “definitely include”) based on the online prioritization survey

Domain	%	Definition
Physical health	95.7	Overall condition of the body at a given time
Psychological health	90.6	Well‐being of mental and emotional state
Physical symptoms	86.5	Departure from normal function or feeling from the body
Mental health	80.2	Cognitive, behavioral, and emotional well‐being
Self‐esteem	75.8	Own worth, ability and value
Pain	71.3	Unpleasant sensory and emotional experience

During the presentation of the online survey on the identified PROs, concerns emerged about how the PROs should be defined. Therefore, the moderator started the day with a discussion about the definition of PROs and which of the PROs participants perceived to be the most important. Afterwards, participants were asked to select and rank their top 3 PROs anonymously.

**FIGURE 1:**
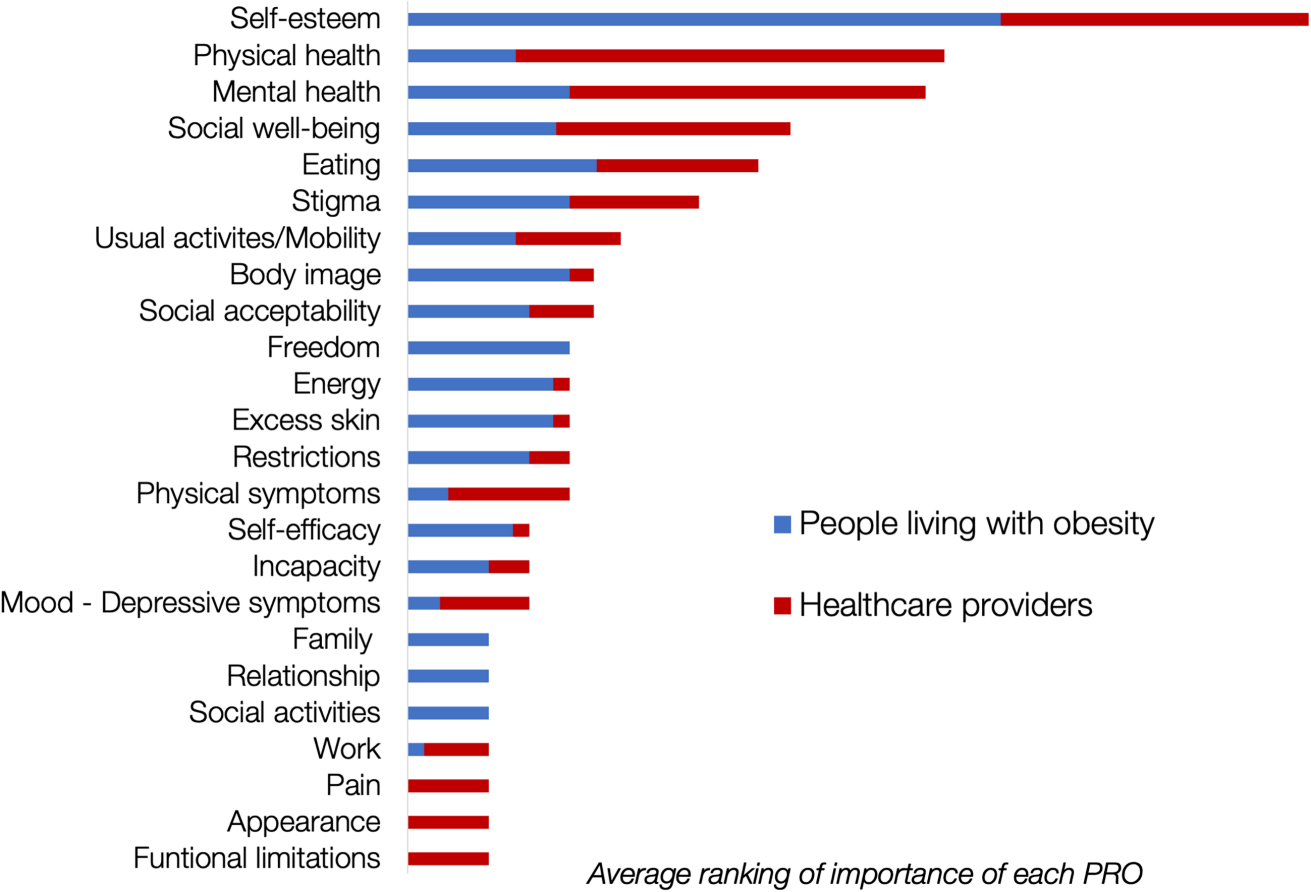
Ranking of the patient‐reported outcomes (PROs) in the face‐to‐face consensus meeting, showing results for people living with obesity and healthcare providers

Self‐esteem was considered the most important PRO, particularly for PLWO, although physical health was perceived to be the most important among HCPs. The voting resulted in the inclusion of the following PROs: self‐esteem, physical health, mental health, social health, stigma, eating, body image, and excess skin (see Figure 1). After the group discussions, participants agreed that the list of PROs was a comprehensive list that captured all PROs relevant to PLWO.

### 3.3 *How* to measure

The second prioritization survey to rank the PROMs was completed by 63 PLWO and 23 HCPs. Only the BODY‐Q and the bariatric and obesity‐specific survey (BOSS) were voted on for inclusion, whereas none of the PROMs were voted upon for exclusion. Therefore, all PROMs were discussed in the consensus meeting (Supporting Information 4). The results of the second survey can be found in Supporting Information 5.

Participants of the consensus meeting voted on PROMs to be included for each specific PRO. The PROMs selected based on face validity and the selected PROMs from the voting rounds are summarized for each PRO in Table 2. For each PRO, one or more PROMs were selected, but no PROM could be selected for stigma due to the lack of PROMs validated with PLWO. The following PROMs were at least once selected: impact of weight on quality of life (IWQOL)‐Lite, Short Form (SF)‐36, BODY‐Q, Obesity‐related Problems (OP)‐Scale, and Quality of Life for Obesity Surgery (QOLOS). No consensus was reached during the meeting on the most adequate PROM for the PROs physical health, social health, body image, and excess skin.

**TABLE 2:** The most important domains and the selected PROMs

Domain	PROM(s) available	PROM(s) selected based on face validity	PROM(s) that were selected after the vote^a^
Self‐esteem	IWQOL‐Lite, IWQOL‐Lite CT, PROS, WHO‐QOL BREF	IWQOL‐Lite, IWQOL‐Lite CT	IWQOL‐Lite
Physical health/functioning/symptoms	BAROS, BODY‐Q, BOSS, BQL‐Index, EQ‐5D‐5L, GIQLI, IWQOL‐Lite, IWQOL‐Lite CT, M‐A QOL QII, OP‐scale, PBOT, PROS, QOLOS, SF‐36, TRIM, WHO‐QOL BREF	BODY‐Q, BOSS, IWQOL‐Lite, IWQOL‐Lite CT, PBOT, QOLOS, SF‐36, TRIM	BODY‐Q, IWQOL‐Lite, SF‐36,
Mental/psychological health	BAROS, BODY‐Q, BQL‐Index, IWQOL‐Lite CT, M‐A QOL QII, SF‐36, TRIM, WHO‐QOL BREF	BODY‐Q, BQL‐Index, IWQOL‐Lite CT, SF‐36	BODY‐Q
Social health	BAROS, BODY‐Q, BOSS, BQL‐Index, EQ‐5D‐5L, GIQLI, IWQOL‐Lite, IWQOL‐Lite CT, M‐A QOL QII, OP‐Scale, PBOT, PROS, QOLOS, SF‐36, TRIM, WHO‐QOL BREF	BODY‐Q, BOSS, BQL‐Index, GIQLI, IWQOL‐Lite, OP‐Scale, SF‐36	BODY‐Q, IWQOL‐Lite, OP‐Scale
Stigma	—	—	—
Eating	BODY‐Q, BOSS, M‐A QOL QII, QOLOS, TRIM	BODY‐Q, BOSS, QOLOS	BODY‐Q
Body image	BODY‐Q, QOLOS	BODY‐Q, QOLOS	BODY‐Q, QOLOS
Excess skin	BODY‐Q, QOLOS	BODY‐Q, QOLOS	BODY‐Q, QOLOS

Abbreviations: BAROS, Bariatric Analysis and Reporting Outcome System; BOSS, bariatric and obesity‐specific survey; BQL Index, Bariatric Quality of Life Index; GIQLI, Gastrointestinal Quality of Life Index; IWQOL‐Lite, Impact of Weight Quality of Life‐Lite; IWQOL‐Lite CT, Impact of Weight Quality of Life‐Lite Clinical Trials; M‐A QoLQII, Moorehead‐Ardelt Quality of Life Questionnaire II; OP‐Scale, Obesity‐related Problems Scale; PBOT, Post Bariatric Outcome Tool; PROS, patient‐reported outcomes in obesity; QOLOS, Quality of Life for Obesity Surgery; SF‐36 Short Form Health Survey 36; TRIM, Treatment Related Impact Measure; WHOQOL‐BREF, World Health Organization Qualitiy of Life Questionnaire‐BREF.

^a^ PROMs were selected if >70% of the participants (people living with obesity and healthcare providers) or >70% of the persons living with obesity selected the PROM for that specific domain.

## 4 DISCUSSION

The goal of the S.Q.O.T. I multi‐professional, international meeting including PLWO was to obtain consensus on PROs (*what* to measure) and PROMs (*how* to measure) to be used in obesity treatment research. Formal presentations, nominal group techniques and modified Delphi exercises were used to develop consensus‐based recommendations.

The results demonstrated that PLWO and HCPs consider different PROs to be the most important. PLWO and HCPs selected eight PROs: self‐esteem, physical health, mental health, social health, eating, stigma, body image, and excess skin. HCPs voted on broad PROs including physical or mental health, while PLWO voted on more specific PROs such as self‐esteem, body image or excess skin. Furthermore, more HCPs voted on symptoms, including depressive symptoms, physical symptoms, and pain. For each PRO, one or more PROMs were selected, but there is currently no validated PROM available to assess obesity stigma. The selected PROs are in line with a previous qualitative study of patient perspectives in persons who had undergone bariatric surgery.^29^ The main differences were that self‐esteem and stigma were not described in the qualitative study, and that the participants from the current study did not vote sexual life to be among the most important PROs.

This was the first consensus meeting that identified which PROs should be collected as a minimum in obesity treatment and how these PROs should be measured, following a rigorous and patient‐centered methodology. We used the previous work of two different COSs developed for obesity treatment research (BARIACT study and STAR‐LITE study) as a starting point. There are, however, some differences with the STAR‐LITE study that are important to mention. In the STAR‐LITE study, “self‐confidence and self‐esteem” were selected separately from QoL in the optional outcome set with a corresponding PROM that was not included in our consensus meeting due to the lack of validation evidence in the treatment of obesity (the Warwick‐Edinburgh Mental Well‐being Scale).^14^ The EQ‐5D‐5L that was recommended to measure QoL in the STAR‐LITE study was not selected in our consensus meeting because it does not capture the PROs considered most important by PLWO.^14^ A next S.Q.O.T. consensus meeting will focus on the selection of one PROM for each PRO, and it should be discussed if items reflecting stigma are represented in other PROMs. If this is not the case, a literature review should be undertaken to identify existing PROMs that measure stigma (e.g., developed for other populations) and whether these can be used in obesity treatment, or such PROMs may need to be developed if none is available. Given that stigma toward PLWO is pervasive,^30^ and the negative impact of experiencing and internalizing weight stigma,^31^, ^32^ there is a need to adequately measure both in obesity treatment research.

A strength of this study is the high number of PLWO that participated. The HCPs included academics from different disciplines and continents. There are no definite guidelines on the sample size of a consensus meeting, but the COMET handbook describes that an adequate number of people attending the in‐person meeting is helpful to fully represent the patient's view.^11^, ^33^ In this consensus meeting, the ratio of PLWO and HCPs was nearly 1:1. This was to reflect the input from PLWO and HCPs equally, which may ensure that the PROs and PROMs chosen are suitable and well accepted. This meeting showed the importance of including PLWO and HCPs, as the selected key PROs were different in these groups.

There are limitations to this study. First, the majority of PLWO were from the United Kingdom and Ireland. The PLWO from the Netherlands had to be fluent in English, through which it was weighted toward higher educated individuals. It is important to note that the HCPs comprised representatives from all continents, except Africa. Second, a few of the participants participated in the development of a PROM that was selected for the consensus meeting. Participants who had a conflict of interest were not excluded, as the number of participants with a conflict of interest was too low to influence the results. Third, the prioritization survey for the selection of PROs lacked relevance due to disagreements by the participants because no clear definitions were given on the PROs and too many different PROs were considered relevant. Therefore, the moderator decided to start the consensus meeting with a group discussion on the definition and selection of PROs. Finally, at the very beginning, the group discussion led to a broad discussion with little consensus. Many domains were deemed important by the different stakeholders. Even though the group discussion was time‐consuming, it was very important for the group dynamic and to reach an agreement on the meaning of the specific PROs. Furthermore, PROs emerged in the group discussion that were not covered in the PROMs identified in the systematic reviews. These PROs would otherwise not have been considered in the voting rounds.

## 5 CONCLUSION

PROs are crucial endpoints in clinical trials and prospective studies of any modality of obesity treatment. To enable data evidence synthesis including outcomes that reflect the views of PLWO, standardized data collection of PROs is key. This consensus meeting was a first step toward standardizing PROs *(what* to measure) and PROMs *(how* to measure) in obesity treatment research. It provides an initial presentation of key PROs and preferred PROMs for obesity treatment research.

## CONFLICT OF INTEREST

None of the members of the organizing committee (C.V., V.M., B.W., I.J., and R.L.) and none of the participants received payment for their participation. Travel expenses and the hotel overnight for the face‐to‐face meeting of the S.Q.O.T. were supported by Medtronic, Johnson & Johnson, Philips Vital Health, Novo Nordisk, Castor and Bart Torensma. C.V. and V.M. are the founders of the S.Q.O.T. initiative, B.W., I.J., and R.L. are cofounders of the S.Q.O.T. initiative. R.C. received lecture, presentations, speakers bureaus, manuscript writing, or educational events fees for Johnson & Johnson, Medtronic, Jansen Pharmaceutical, Novo Nordisk, BAriatec, and GI Dynamics; a research grant from Medtronic; consulting fees from Johnson & Johnson and Medtronic. X.R.S. received lecture, presentations, speakers bureaus, manuscript writing, or educational events fees for Obesity Canada, the European Association for the Study of Obesity, and the World Health Organization Regional Office for Europe; conference travel fees from Obesity Canada, the European Association for the Study of Obesity, the World Health Organization Regional Office for Europe, World Obesity Federation, The Obesity Society, and Novo Nordisk consulting fees from Obesity Canada, the European Association for the Study of Obesity, and the World Health Organization Regional Office for Europe; is co‐chair of the World Obesity Federation Working Group on Weight Bias and Stigma, member of The Obesity Society Policy and Advocacy Committee, and founding member of the Obesity Canada EveryBODY Matters Collaborative (unpaid); owns a research and communications company through which she conducts her consulting work (K&X Ramos AB). R.L. received lecture, presentations, speakers bureaus, manuscript writing, or educational events fees for Johnson & Johnson, Medtronic. S.F. received academic conference attendance fees from Johnson & Johnson and Novo Nordisk. B.B. received from Monash University funding support for attendance, as the main account holders of the funding received from the Department of Health, Commonwealth of Australia. Monash University did not provide any additional funding or financial support for the attendance outside of the grant funding money as explained above. W.B. received lecture, presentations, speakers bureaus, manuscript writing, or educational events fees for Merck Sharpe and Dohme, Novo Nordisk, and Gore; research grants from Medtronic, Johnson & Johnson, Gore, Applied Medical, Novo Nordisk, and Commonwealth Government of Australia; conference travel fees from Novo Nordisk. All other authors declare that they have no conflict of interest.

## Supporting information


**Figure S1:** Flow diagram of search
**Figure S2:** Results of the online survey assessing which domains that should be included in QoL measurement
**Table S1:** Characteristics of the included studies

## References

[1] Yumuk V , Tsigos C , Fried M , et al. European guidelines for obesity management in adults. Obes Facts. 2015;8(6):402‐424. doi:10.1159/000442721 26641646 PMC5644856

[2] Bray GA , Fruhbeck G , Ryan DH , Wilding JP . Management of obesity. Lancet (London, England). 2016;387(10031):1947‐1956. 10.1016/S0140-6736(16)00271-3 26868660

[3] Welbourn R , Hollyman M , Kinsman R , et al. Bariatric surgery worldwide: baseline demographic description and one‐year outcomes from the fourth IFSO global registry report 2018. Obes Surg. 2019;29(3):782‐795. doi:10.1007/s11695-018-3593-1 30421326

[4] Brethauer SA , Kim J , el Chaar M , et al. Standardized outcomes reporting in metabolic and bariatric surgery. Surg Obes Relat Dis. 2015;11(3):489‐506. doi:10.1016/j.soard.2015.02.003 26093765

[5] Coulman KD , Abdelrahman T , Owen‐Smith A , Andrews RC , Welbourn R , Blazeby JM . Patient‐reported outcomes in bariatric surgery: a systematic review of standards of reporting. Obes Rev. 2013;14(9):707‐720. doi:10.1111/obr.12041 23639053

[6] Martenstyn J , King M , Rutherford C. Impact of weight loss interventions on patient‐reported outcomes in overweight and obese adults with type 2 diabetes: a systematic review. J Behav Med. 2020;43(6):873‐891. doi:10.1007/s10865-020-00140-7 32060765

[7] Patrick DL , Burke LB , Powers JH , et al. Patient‐reported outcomes to support medical product labeling claims: FDA perspective (formerly with FDA); 4 Mapi Values Ltd, Cheshire, UK (formerly with FDA); why a Guidance on Patient‐Reported Outcomes (PROs)? Value Heal. 2007;10:S125‐S137. 10.1111/j.1524-4733.2007.00275.x 17995471

[8] de Vries CEE , Kalff MC , Prinsen CAC , et al. Recommendations on the most suitable quality‐of‐life measurement instruments for bariatric and body contouring surgery: a systematic review. Obes Rev. 2018;19(10):1395‐1411. doi:10.1111/obr.12710 29883059

[9] COMET Initiative . |Available from: https://www.comet-initiative.org/

[10] ICHOM . ICHOM Standard Sets. Available from: https://www.ichom.org/standard-sets/

[11] Williamson PR , Altman DG , Bagley H , et al. The COMET Handbook: version 1.0. Trials. 2017;18(3):1‐50. 28681707 10.1186/s13063-017-1978-4 PMC5499094

[12] Williamson PR , Altman DG , Blazeby JM , et al. Developing core outcome sets for clinical trials: issues to consider. Trials. 2012;13:132. 22867278 10.1186/1745-6215-13-132 PMC3472231

[13] Coulman KD , Hopkins J , Brookes ST , et al. A core outcome set for the benefits and adverse events of bariatric and metabolic surgery: the BARIACT Project. PLoS Med 2016; 13(11):e1002187. doi:10.1371/journal.pmed.1002187 27898680 PMC5127500

[14] Mackenzie RM , Ells LJ , Simpson SA , Logue J . Core outcome set for behavioural weight management interventions for adults with overweight and obesity: standardised reporting of lifestyle weight management interventions to aid evaluation (STAR‐LITE). Obes Rev. 2020;21(2):1‐25. 10.1111/obr.12961 PMC7050499 31756274

[15] WHOQOL ‐ Measuring Quality of Life . |Available from: https://www.who.int/tools/whoqol

[16] Prinsen CAC , Mokkink LB , Bouter LM , et al. COSMIN guideline for systematic reviews of patient‐reported outcome measures. Qual Life Res. 2018 May;27(5):1147‐1157. doi:10.1007/s11136-018-1798-3 PMC5891568 29435801

[17] Aasprang A , Våge V , Flølo TN , et al. Patient‐reported quality of life with obesity—development of a new measurement scale. Tidsskr nor Laegeforen. 2019;139(11). 10.4045/tidsskr.18.0493 31429227

[18] Qualtrics . 2005. Provo, Utah, USA. Available from: https://www.qualtrics.com

[19] Diamond IR , Grant RC , Feldman BM , et al. Defining consensus: a systematic review recommends methodologic criteria for reporting of Delphi studies. J Clin Epidemiol. 2014;67(4):401‐409. doi:10.1016/j.jclinepi.2013.12.002 24581294

[20] Vogel C , Zwolinsky S , Griffiths C , Hobbs M , Henderson E , Wilkins E. A Delphi study to build consensus on the definition and use of big data in obesity research. Int J Obes (Lond). 2019;43(12):2573‐2586. doi:10.1038/s41366-018-0313-9 30655580 PMC6892733

[21] Wilson IB , Cleary PD . Linking clinical variables with health‐related quality of life. A conceptual model of patient outcomes. JAMA. 1995;273(1):59‐65. 7996652

[22] Intro to PROMIS. Available from: https://www.healthmeasures.net/explore-measurement-systems/promis/intro-to-promis

[23] Cella D , Yount S , Rothrock N , et al. The Patient‐Reported Outcomes Measurement Information System (PROMIS): progress of an NIH roadmap cooperative group during its first two years. Med Care. 2007;45(5 SUPPL. 1):S3‐S11. 10.1097/01.mlr.0000258615.42478.55 PMC2829758 17443116

[24] Jones J , Hunter D . Consensus methods for medical and health services research. BMJ [Internet]. 1995;311(7001):376‐380. 10.1136/bmj.311.7001.376 PMC2550437 7640549

[25] RAND . Delphi method. Available from: https://www.rand.org/topics/delphi-method.html

[26] McMillan SS , King M , Tully MP . How to use the nominal group and Delphi techniques. Int J Clin Pharmacol. 2016;38(3):655‐662. 10.1007/s11096-016-0257-x PMC4909789 26846316

[27] VoxVote . 2019. Available from: https://www.voxvote.com/

[28] Terwee CB , Prinsen CAC , Chiarotto A , et al. COSMIN methodology for evaluating the content validity of patient‐reported outcome measures: a Delphi study. Qual Life Res. 2018;27(5):1159‐1170. doi:10.1007/s11136-018-1829-0 29550964 PMC5891557

[29] Coulman KD , MacKichan F , Blazeby JM , Donovan JL , Owen‐Smith A. Patients' experiences of life after bariatric surgery and follow‐up care: a qualitative study. BMJ Open. 2020;10(2):e035013. doi:10.1136/bmjopen-2019-035013 PMC7045271 32034030

[30] Flint SW . Time to end weight stigma in healthcare. EClinicalMedicine. 2021;34:100810. 33870153 10.1016/j.eclinm.2021.100810 PMC8042345

[31] Täuber S , Gausel N , Flint SW . Weight bias internalization: the maladaptive effects of moral condemnation on intrinsic motivation. Front Psychol. 2018;9:1836. 30319517 10.3389/fpsyg.2018.01836 PMC6170635

[32] Sutin AR , Stephan Y , Terracciano A. Weight discrimination and risk of mortality. Psychol Sci. 2015;26(11):1803‐1811. doi:10.1177/0956797615601103 26420442 PMC4636946

[33] Murphy MK , Black NA , Lamping DL , et al. Consensus development methods, and their use in clinical guideline development. Heal Technol Assess. 1998;2(3):1‐88. 9561895

