# Supplementary material for: Outcomes of the first global multidisciplinary consensus meeting including persons living with obesity to standardize patient‐reported outcome measurement in obesity treatment research
Source: Obes Rev. 2022 May 29;23(8):e13452. doi: 10.1111/obr.13452 (PMC9539945; doi:10.1111/obr.13452)
Supplement: Supplementary file 1 — Figure S1: Flow diagram of search Figure S2: Results of the online survey assessing which domains that should be included in QoL measurement Table S1: Characteristics of the included studies [file OBR-23-e13452-s001.pdf]

Supporting Information 1: Details of systematic review

Eligibility criteria

All instruments developed for patients with obesity undergoing any type of treatment were eligible. Only studies with full text papers and with the aim to describe the development and/or evaluation of measurement properties of instruments that measure quality of life were included. Since the consensus meeting was held in English only instruments available in the English language were used for this review.

Literature search

On 22 april 2019, a systematic literature search was conducted in PubMed, EMBASE, Ebsco/PsycINFO, Ebsco/CINAHL, Cochrane Database Systematic Reviews and CENTRAL. The search included, but was not limited to the following terms:

- Obesity
- Patient-reported outcome measures
- Quality of Life
- Lifestyle intervention
- Nutrition
- Movement therapy
- Cognitive behavioral therapy
- Pharmacological treatment
- Endoscopic treatment
- Clinimetrics/psychometrics

Using Covidence systematic review software (Veritas Health Innovation, Melbourne, Australia. Available at www.covidence.org) two reviewers (CV and VM) independently screened titles and abstracts and, at a second stage, assessed the full-text articles retrieved by the literature search. Conflicts were resolved by consensus of the two reviewers.

Evaluation of methodological quality

The same two reviewers (CV and VM) independently evaluated the methodological quality of included studies. The COSMIN (COnsensus-based Standards for the selection of health Measurement INstruments) guideline for systematic reviews of measurement instruments was used to evaluate the methodological quality of the included studies (1). Conflicts were resolved by consensus of the two reviewers. For each included instrument development studies were searched to complete quality evaluation.

Since one reviewer (CV) worked in the department of one of the included instruments (the BODY-Q), this instrument was rated by another reviewer (MN).

Selection of instruments

The previous review included 26 articles with 24 instruments (2). After exclusion of instruments focused on body contouring surgery (n=1) and instruments in other languages then English (n=12), a total of 11 instruments could be used in the consensus meeting. Studies on development and/or evaluation of measurement properties of these 11 instruments were described in 14 publications.

The updated search resulted in seven additional instruments, two of these instruments were not available in English and hence not included (3,4).

In addition, one instrument was brought to our attention via a of the member of the consensus meeting panel (5).

Figure 1: Flow diagram of search.

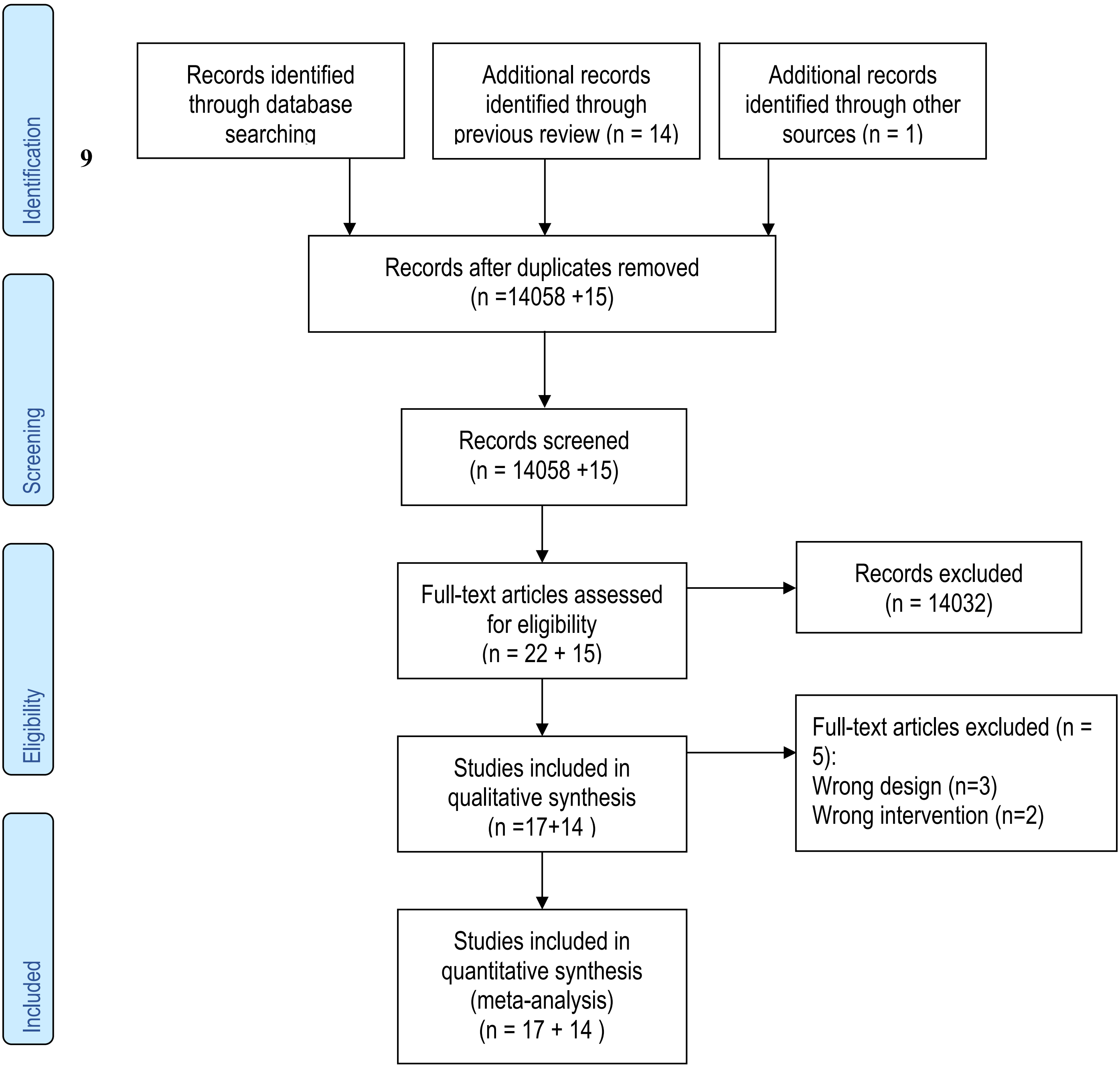

Table 1: Characteristics of the included studies

Instrument	author	Year of publication	Geographic location(s)	Language	Population	Number of participants	Age, years	Percentage of women	BMI, kg m^2
M-A QoLQ (BAROS)	Oria HE	1998	US	English	n.a.	n.a.	n.a.	n.a.	n.a.
BOSS	Tayyem RM	2014	UK	English	Pre and postbariatric patients	236	45.3±10.7	77.1%	48.4±9.2
Laval	Donini	2017	Italy	Italian	Patients in treatment for obesity	273	46.2 ± 14.2 (m)<br>46 ± 13.5 (f)	72,9%	40.4 ± 8.3 (M) 34.8 ± 6.2 (F)
TRIM	Brod et al	2010	US, Australia, and Canada	English	Patients who use anti-obesity medication	208	20-76 years	78.4%	30-45
SF-36	Corica	2016	Italy	Italian	obese subjects seeking treatment	1735	44.7 ± 11.0	77.6%	30-45

IWQOL-lite	Kolotkin	1997							
	De Mariano	2010	Brazil	Portugese	Premenopausal women in weight loss program (excluded: chronic diseases physical disabilities and smokers.)	89 clinical<br><br>156 community	36.0 (±7.8) (clinical)<br><br>34.0 (±7.6) community	?	29.3 ±5.3 (clinical)<br><br>24.4 ±5.0 community
	Engel	2005	Portugal	Portugese	Outpatient lifestyle weight management programme & overweight/obese volunteers (all women)<br>exclu: pre-menopausal, free from	138 clinical<br>250 community			

					current major chronic and without limiting physical disability.				
IWQOL-lite Clinical trial version	Kolotkin	2017	US	English	Pts with obesity only	42	19-70	52.4%	30.4-51.6
					Pts with obesity and diabetes	29	21-75	16/29	27.1–45.7
Obesity-related Problems scale	Karlsson	2003	Sweden	Swedish	Obese subjects	6863	37-57	4264	
	Karlsson	1995	Sweden	Swedish		709	47-48 per group on average	312	
Moorhead-Ardelt Quality of life Questionnaire II	Oria	2009	US	English					

QOLOD	Ziegler	2005	France	French	Pts with obesity, excluded those with obesity of endocrine origin	128 & 212	42.5 ± 12.1 & 43.3 ± 12.2	83.6% & 77.7%	34.5 ± 2.8 & 35.8 ± 7.5

References

1. Prinsen CAC, Mokkink LB, Bouter LM, Alonso J, Patrick DL, de Vet HC, et al. COSMIN guideline for systematic reviews of patient-reported outcome measures. Qual Life Res. 2018 May;27(5):1147-1157.
2. de Vries CEE, Kalff MC, Prinsen CAC, Coulman KD, den Haan C, Welbourn R, et al. Recommendations on the most suitable quality-of-life measurement instruments for bariatric and body contouring surgery: a systematic review. Obes Rev. 2018;19(10):1395–411.
3. Sartorio A, Agosti F, De Col A, Castelnuovo G, Manzoni GM, Molinari E, et al. Concurrent comparison of the measurement properties of generic and disease-specific questionnaires in obese inpatients. J Endocrinol Invest. 2014 Jan;37(1):31-42.
4. Ziegler O, Filipecki J, Girod I, Guillemin F. Development and validation of a French obesity-specific quality of life questionnaire: Quality of Life, Obesity and Dietetics (QOLOD) rating scale. Diabetes Metab. 2005 Jun;31(3 Pt 1):273-83.
5. Aasprang A, Våge V, Flølo TN, Hegland PA, Kolotkin R, Natvig GK, et al. Patient-reported quality of life with obesity - development of a new measurement scale. Tidsskr Nor Laegeforen. 2019 Aug 19;139(11).

Supporting Information 2: Prioritization surveys

Prioritization survey 1:

Please indicate for each domain if you think this domain should be definitively included, possibly included or definitively excluded in quality of life measurement for obesity treatment.

	Definitively include	Maybe include	Definitively exclude
Appearance			
Physical Health			
Physical Symptoms			
Psychological Health			
Sexual Well-being			
Social Health			
Body Image			
Self Esteem			
Work Function			
Eating			
Incapacity			
Personal Hygiene			
Emotional distress			
Anxiety			
Pain			
Digestive symptoms			
Family			

	Definitively include	Maybe include	Definitively exclude
Positive activities			
Partnership			
Excess skin			
Usual activities			
Self care			
Fatigue			
Mental Health			
Self-Efficacy			

Is there a domain that is not in the list, but should be according to you?

Thank you for filling in the survey!

Prioritization survey 2:

Dear Participant,

Thank you for assisting the SQOT initiative to rank PROMs that measure Quality of Life in obesity. As explained in the e-mail, this survey will be used as a basis for the consensus meeting on Quality of Life measurement in obesity treatment.

Recently, our team performed a systematic review on PROMs in surgery for obesity. We will ask you the rate the PROMs included in that survey.

At the end of the survey you will be able to add additional instruments that are not listed in the survey. Please add the instrument along with why you think that it is important.

For each PROM (ordered based on their category of recommendation in the systematic review by de Vries et al.):

Categorization of this PROM:

- Definitively include
- Possibly include
- Definitively exclude

Comment:

Supporting Information 3: Prioritization surveys: ranking of domains

Figure 1: Results of the online survey assessing which domains that should be included in QoL measurement

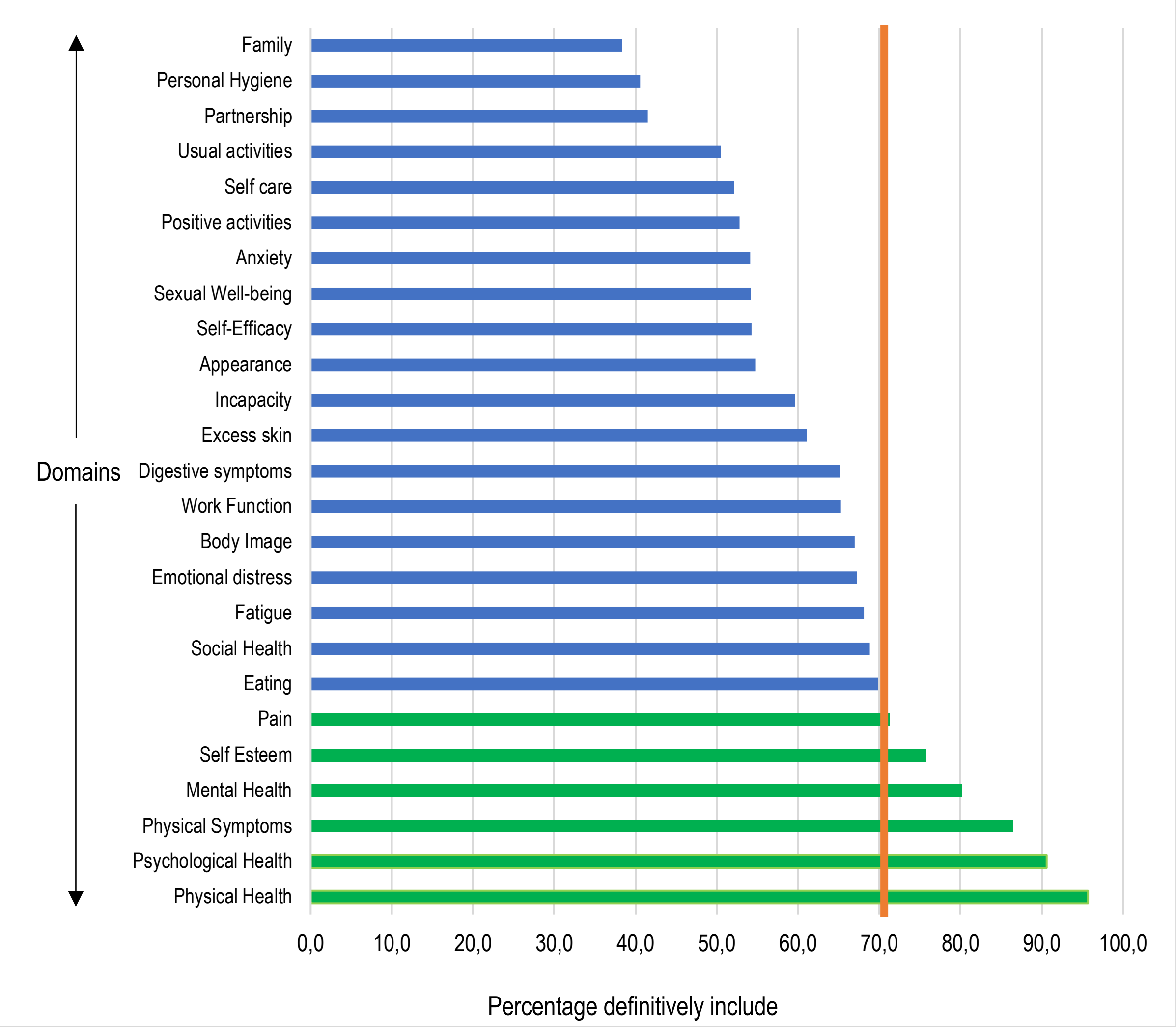

Supporting Information 4: PROMs included in the consensus meeting

1. BAROS
2. BODY-Q
3. BOSS
4. BQL-Index
5. EQ-5D-5L
6. GIQLI
7. IWQOL-Lite
8. IWQOL-Lite CT
9. M-A QOL QII
10. OP-scale
11. ORWELL-97
12. PBOT
13. PROS
14. QOLOS
15. SF-36
16. TRIM
17. WHO-QOL BREF

BAROS, Bariatric Analysis and Reporting Outcome System; BOSS, bariatric and obesity-specific survey; BQL Index, Bariatric Quality of Life Index; GIQLI, Gastrointestinal Quality of Life Index; IWQOL-Lite, Impact of Weight Quality of Life-Lite; M-A QoLQ, Moorehead-Ardelt Quality of Life Questionnaire; M-A QoLQII, Moorehead-Ardelt Quality of Life Questionnaire II; OP-scale, Obesity-related Problems scale; ORWELL-97, Obesity-Related WELL-being-97; PBOT, Post Bariatric Outcome Tool; PROS, Patient-Reported Outcomes in Obesity; QOLOS, Quality of Life for Obesity Surgery; SF-36, Short-Form-36; TRIM,

Treatment Related Impact Measure; WHO-QOL BREF, World Health Organization Qualitiy of Life Questionnaire-BREF

Supporting Information 5: Ranking of PROMs in the first prioritization survey

PROM	Survey option	Percentage, %
BAROS	Definitely include<br>Possibly include<br>Definitely exclude	38<br>17<br>45
BODY-Q – Domain:<br>Quality of Life	Definitely include<br>Possibly include<br>Definitely exclude	77<br>17<br>6
BODY-Q – Domain:<br>Appearance	Definitely include<br>Possibly include<br>Definitely exclude	56<br>26<br>18
BOSS	Definitely include<br>Possibly include<br>Definitely exclude	73<br>22<br>4
BQL-Index	Definitely include<br>Possibly include<br>Definitely exclude	35<br>33<br>33
EQ-5D-5L	Definitely include<br>Possibly include<br>Definitely exclude	38<br>38<br>24
GIQLI	Definitely include<br>Possibly include<br>Definitely exclude	43<br>28<br>30
IWQOL-Lite	Definitely include<br>Possibly include<br>Definitely exclude	59<br>31<br>10
M-A QOL QII	Definitely include<br>Possibly include<br>Definitely exclude	34<br>29<br>37
OP-scale	Definitely include<br>Possibly include<br>Definitely exclude	43<br>30<br>27

PBOT	Definitely include<br>Possibly include<br>Definitely exclude	59<br>28<br>14
QOLOS	Definitely include<br>Possibly include<br>Definitely exclude	64<br>23<br>13

BAROS, Bariatric Analysis and Reporting Outcome System; BOSS, bariatric and obesity-specific survey; BQL Index, Bariatric Quality of Life Index; GIQLI, Gastrointestinal Quality of Life Index; IWQOL-Lite, Impact of Weight Quality of Life-Lite; M-A QoLQ, Moorehead-Ardelt Quality of Life Questionnaire; M-A QoLQII, Moorehead-Ardelt Quality of Life Questionnaire II; OP-scale, Obesity-related Problems scale; PBOT, Post Bariatric Outcome Tool; QOLOS, Quality of Life for Obesity Surgery
